# Ex Vivo Evaluation of CD3^+^CD8^+^ T Cell Subpopulations in Red Blood Cell Concentrates: Does Storage Time Play an Important Role?

**DOI:** 10.3390/jcm15031178

**Published:** 2026-02-03

**Authors:** Salih Haldun Bal, Levent Tufan Kumas, Lacin Cevhertas, Izel Yilmaz, Pinar Hiz-Ellergezen, Ferah Budak-Sener, Yasemin Heper, Haluk Barbaros Oral

**Affiliations:** 1 Department of Immunology, Faculty of Medicine, Bursa Uludağ University, 16059 Bursa, Türkiye; fbudak@uludag.edu.tr (F.B.-S.); oralb@uludag.edu.tr (H.B.O.); 2 Dr. Raşit DURUSOY Blood Bank, Faculty of Medicine, Bursa Uludağ University, 16059 Bursa, Türkiye; ltkumas@uludag.edu.tr (L.T.K.); yheper@uludag.edu.tr (Y.H.); 3 Department of Medicine-Immunology, Institute of Health Science, Bursa Uludağ University, 16059 Bursa, Türkiye; lacincevhertas@gmail.com (L.C.); izelyilmaz91@gmail.com (I.Y.); pinarhiz@gmail.com (P.H.-E.); 4 Department of Infectious Diseases and Clinical Microbiology, Faculty of Medicine, Bursa Uludağ University, 16059 Bursa, Türkiye

**Keywords:** CD3^+^CD8^+^ T cell subpopulations, red blood cell concentrate, storage time

## Abstract

**Background/Objectives**: Our study was designed to explore the potential role of allogeneic CD8^+^ T lymphocytes present in red blood cell concentrates (RBCs) in the development of transfusion-related immunomodulation (TRIM) and the effect of storage time on these cells. **Methods**: From six units of whole blood, donated by volunteers, RBCs were obtained and each one was divided into three equal parts to provide the samples for storage days 0, 21, and 42. On related days, mononuclear cells (MNCs) were isolated from these RBC samples. MNCs were cultured, and phytohemagglutinin was added to half of the culture wells to stimulate the cells and achieve T cell division. Supernatants and MNCs were obtained from stimulated (STI) and unstimulated (US) wells. Supernatants were used for cytokine analyses, while MNCs were used to investigate the T cells and transcription factors. **Results**: The frequency of CD8^+^ T lymphocytes (Tc), their subgroups (Tc1, Tc2, and Tc17), specific transcription factors, and effector cytokines decreased during the storage time, but cell viability increased. CD3^+^CD8^+^TNF-α+ cells were significantly higher in the STI group on day 0 compared to the US group. Other cells did not respond to the mitogen (phytohemagglutinin) stimulation. **Conclusions**: During storage, the number of Tc cells and their ability to respond to mitogens decreased over time. The unresponsiveness was not recovered in ex vivo cell culture. Our findings suggest that transfused Tc cells are unlikely to be primary mediators of TRIM.

## 1. Introduction

Transfusion-related immunomodulation (TRIM) is a hazardous complication of allogeneic blood transfusion (ABT). TRIM was first defined based on the observation that allogeneic blood transfusions prolong graft survival in patients awaiting kidney transplantation [1]. TRIM has also been associated with clinical conditions such as an increase in cancer recurrence, in postoperative bacterial infections, and in short-term mortality rates and a decrease in Crohn’s disease recurrence, in recurrent spontaneous abortions, and in the reactivation of certain latent infections (CMV, HIV, etc.) [2]. TRIM can be defined as changes in the immune system of the ABT recipient. It is presumed that these changes are responsible for the clinical outcome of TRIM. Some of these immunological changes are the suppression of T cell proliferation, decreased T cell count, decrease in the CD4^+^/CD8^+^ ratio, and polarisation of the immune response from T helper 1 (Th1) to Th2 [3,4,5,6]. Allogeneic blood cells and molecules that accumulate in the blood component during storage are believed to be triggers of TRIM [2]. Furthermore, transfusion of long-stored blood components (older than 35 storage days) and the number of transfused components also enhance the adverse transfusion effects [7,8,9]. In addition, many variables can complicate the clinical situation, such as the age, comorbidity, and immune status of the patients, the variety of products, and the timing of transfusion [10]. However, to date, studies about ABT’s immunomodulatory effects and the mechanisms which are triggering TRIM have been insufficient.

TRIM cannot be diagnosed quickly by symptoms like other transfusion complications; long-term clinical outcomes are important. This makes TRIM difficult to diagnose and detect. Information about some special contents of blood components could be important for understanding the immunomodulatory aspects that may influence TRIM. We hypothesise that the factors that trigger TRIM in the ABT recipient may also have a similar effect within the blood component. In addition, increased kidney allograft survival, cancer recurrence, and viral infection recurrence may indicate that ABT suppresses CD8^+^ T lymphocytes in the recipient, as studies support [11]. For this reason, our objective was to evaluate CD8^+^ T cells in the blood bag. Our main purpose was to investigate whether the suppression of CD8^+^ T cells begins within red blood cell concentrates (RBCs). We also analysed the relationship between the CD8^+^ T cells and the storage time of RBCs. Therefore, ex vivo analysis of CD8^+^ cells, their subgroups, specific transcription factors, and effector cytokines was performed in MNC cultures.

## 2. Materials and Methods

### 2.1. Preparation of Blood Components

**Figure 1:**
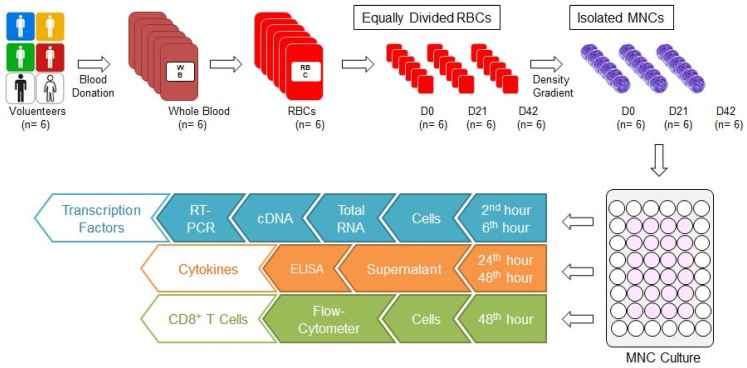
Study algorithm. Six units of whole blood were donated and six units of RBCs were obtained. Each RBC was divided into 3 equal parts and RBC samples for D0, D21, and D42 were prepared. The MNCs were isolated from each one using the density gradient method, and cultured. Cells were collected in the second and sixth hours from the culture wells for analysis of transcription factors. First, total RNA was isolated, then cDNA was synthesised, and RT-PCR was performed. Culture media were collected from culture wells at the 24th and 48th hours for cytokine analysis. Cytokine concentrations were analysed by ELISA. Cells were collected from culture wells at the 48th hour for CD8^+^ T cell analysis and evaluated by FC. cDNA: Complementary DNA; D0: Day 0; D21: Day 21; D42: Day 42; ELISA: Enzyme-linked immunosorbent assay; FC: Flow-cytometer; MNC: Mononuclear cell; RBC: Red blood cell concentrate; RT-PCR: real-time polymerase chain reaction.

Our study was conducted with the approval of the ethics committee of Bursa Uludag University Faculty of Medicine (No: 2015-12/26). Six units of whole blood (each one 450 ± 10 mL) were collected from six male donors aged between 35 and 45 who are compatible with the national blood donor selection criteria. Six units of non-leukoreduced RBCs with additive solution (SAG-M) were supplied from six units of whole blood through Reveos Automated Blood Processing System (Terumo, Lakewood, CO, USA) in our hospital blood bank. Each RBC was divided into 3 equal parts to prepare 0, 21, and 42 storage day samples (D0, D21, and D42, respectively) (Figure 1). While the D0 sample was prepared for the isolation of mononuclear cells (MNCs), the samples D21 and D42 were stored at +4 °C in the blood bank refrigerators (Nüve, Ankara, Türkiye) until day 21 and 42, for isolation of MNC. Because the storage time for RBCs with SAG-M are 42 days, first (D0), middle (D21), and last (D42) storage days were selected for the investigation of the effects of storage time.

### 2.2. Mononuclear Cell Culture

Mononuclear cells (MNCs) were isolated from RBCs by density gradient method (Lymphocyte Separation Medium, Capricorn Scientific; GmbH, Ebsdorfergrund, Germany) (Lymphoprep, Axis-Shield PoC AS; Oslo, Norway) (Figure 1). MNC were seeded at 5 × 10^5^ cells/well in 48-well plates (Costar 3548; Corning Incorporated, NY, New York, USA) within 500 µL of complete RPMI [RPMI 1640 with L-Glutamine, L-Glutamine, Penicillin/Streptomycin, FBS (Lonza; Verviers, Belgium), MEM vitamin (Wisent Inc.; Quebec, Canada), Kanamycin, Na-Pyruvate/Non essentials AS, Beta-Mercaptoethanol; Gibco, ThermoFisher Scientific, Waltham, MA, USA)]. Subsequently, 1 µL mitogen (a substance that stimulates mitosis; phytohemagglutinin; PHA; Merck; Darmstadt, Germany) was added to half of the wells to obtain stimulated (STI) cell culture wells. The rest of the wells remained unstimulated (US). All plates were cultured at 37 °C in the CO_2_ incubator (Panasonic, Tokyo, Japan).

### 2.3. Analyses of Transcription Factors

Specific transcription factors for CD8^+^ T cell subgroups (TBX21, GATA3, RORC2) were analysed with real-time polymerase chain reaction (RT-PCR) (Table 1) [12,13]. Cells were collected at the 2nd and 6th hour from MNC culture wells (Figure 1). Total RNA was isolated using a commercial kit (MO BIO Laboratories Inc., Carlsbad, CA, USA). Complementary DNA (cDNA) was synthesised with the commercial kit (First Strand cDNA Synthesis Kit, New England BioLabs Inc., Ipswich, MA, USA) and stored in a freezer (−20 °C) until RT-PCR analysis. The expression of specific transcription factors was measured using RT-PCR panels (Real-Time Ready, Roche, Mannheim, Germany) designed for this study using Light-Cycler 480 RT-PCR (Roche, Mannheim, Germany). Panels consisted of three genes related to the transcription factors (TBX21, GATA3, RORC2), five reference genes (HPRT1, RPL13A, ACTB, GAPDH, YWHAZ), and negative controls. The RT-PCR process consisted of denaturation (1 cycle, 95 °C, 10 min), amplification (45 cycles, 95 °C, 10 s; 60 °C, 30 s; 72 °C, 1 s), and cooling (40 °C, 30 s) steps. Following RT-PCR, relative quantification of target gene expression was performed, and all data were analysed using the ΔΔCt method.

### 2.4. Preparation of Cytokine Samples from MNC Cultures

Culture media were collected from US and STI wells at 24 and 42 h (Figure 1). The media were centrifuged in 3500× *g* for 10 min, then 15,000× *g* for 7 min, and supernatants were obtained. These supernatants were transferred to clean tubes and stored at −80 °C (Revco, Thermo Fisher Scientific, Waltham, MA, USA).

### 2.5. The Analysis of Cytokine Levels

The effector cytokines of the CD8^+^ T cell subgroups (IL4, IL13, IL17, TNF-α, IFN-γ) were selected for analysis (Table 1) [12,13]. The supernatants were thawed after sample collections were completed. Cytokine concentrations were analysed by enzyme-linked immunosorbent assay (ELISA; BioLegend; San Diego, CA, USA) according to the manufacturer’s recommendations. Absorbance was read on a spectrophotometer (Sunrise; Tecan, Switzerland).

### 2.6. Analysis of T Lymphocytes

**Figure 2:**
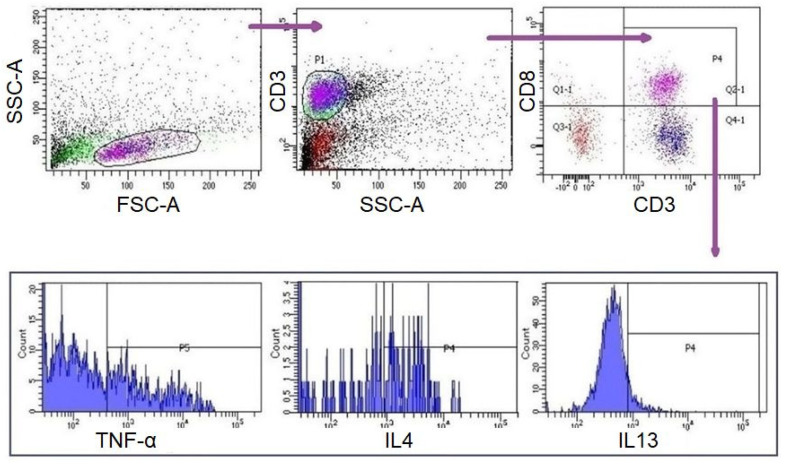
Gating strategy for CD3^+^CD8 ^+^T cells and subgroups. Lymphocytes were initially gated by forward scattering (FSC) and side scattering (SSC) properties. T lymphocytes were gated by SSC and surface expression of CD3^+^. CD8^+^ T lymphocytes and their subgroups were gated and assessed surface expressions and intracellular cytokine expressions were evaluated.

A measurement of 10 µL of PMA/Ionomycin (Santa Cruz; Dallas, Texas, USA) was added to each well of the culture plate at the 42nd hour of incubation. Four hours later (46th hour), 0,5 µL of Brefeldin A (eBioscience; Waltham, MA, USA) was added to each well. Subsequently, cells were collected from the US and STI wells at the 48th hour (Figure 1). CD8^+^ T lymphocytes in these samples were analysed with flow-cytometer (FC) (FACS CANTO II, BD Biosciences, San Jose, CA, USA) and analysed by BD FACSDiva Software (BD Biosciences). Fluorescent-labelled monoclonal antibodies (mAbs) were used for FC analyses [IgG1-APC, IgG1-PE, IgG2a-PE, IgG1-Af647/CD3-FITC, CD3-APC, IL4-APC, IL21-APC, IL5-PE, IFNγ-PE, TNFα-PE, IL17-Af647/CD3-FITC (BioLegend; San Diego, CA, USA), IgG1-FITC, CD8-FITC, IL13-FITC (eBioscience); IgG1-PerCP, CD3-PerCP, CD8-PerCP (BD Bioscience)]. For surface staining, cells were labelled with mAbs for 15 min at room temperature (RT) in the dark and were washed with Cell WASH solution (BD Biosciences). Subsequently, intracellular staining was performed for the analysis of T cell subgroups (Table 1) [12,13,14]. To do this, Permeabilizing Solution-2 (BD Biosciences) was added to each test tube and incubated at RT in the dark for 10 min. The samples were then washed with Cell WASH solution and mAbs were added for intracellular staining. The tubes were then incubated again for 30 min at RT in the dark. The samples were washed again with Cell WASH solution and analysed via FC for the expressions of surface markers and intracellular cytokines. Gating strategy and evaluations were carried out as demonstrated in Figure 2. The subgroups of CD3^+^CD8^+^ cells were evaluated in CD3^+^CD8^+^ gate. For viability, cells were incubated with 1 µL of propidium iodide (BioLegend, San Diego, CA, USA) for 10 min at RT in the dark and analysed with FC (FACS CANTO II).

### 2.7. Statistical Analysis

Descriptive statistics are given as median (minimum–maximum) for continuous variables. For the categorical data, descriptive statistics are reported as frequency and percentage. Mann–Whitney U and Wilcoxon signed rank tests were used to compare two groups. Data analysis was performed using IBM SPSS Statistics v.21 software. The significance level was established as α = 0.05.

## 3. Results

### 3.1. CD3^+^CD8^+^ T Lymphocytes

**Figure 3:**
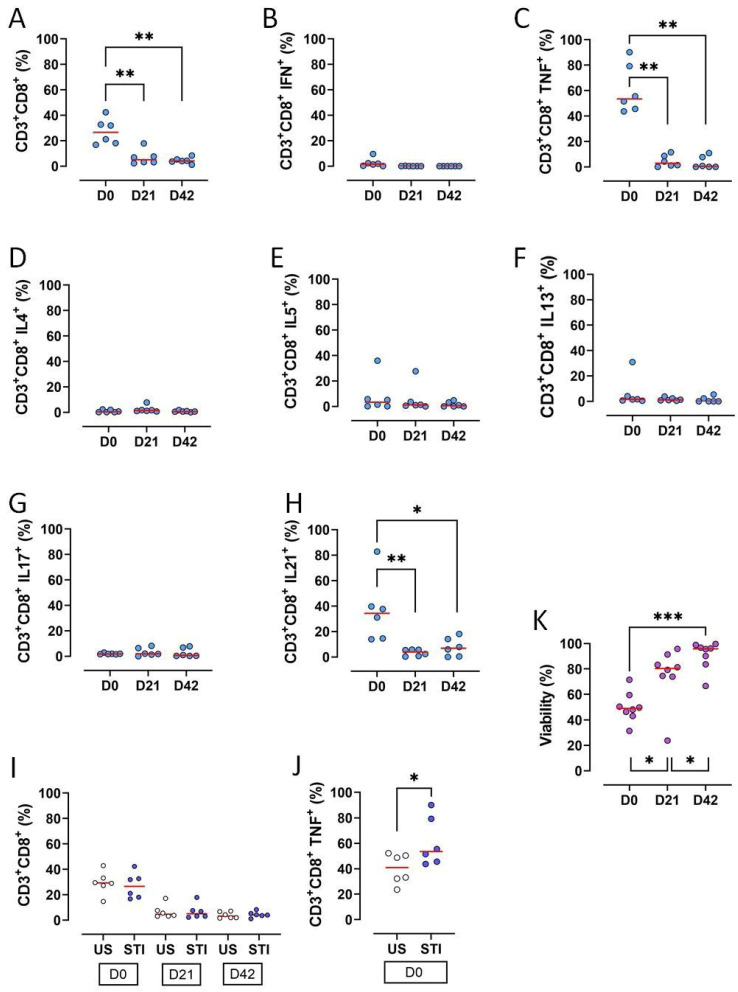
Results and statistical evaluation of CD3^+^CD8^+^ T lymphocytes, their subgroups, and total viability. (**A**) CD3^+^CD8^+^ T cells decreased significantly on D21 and D42 compared to D0. (**B**) IFN-γ expressing CD3^+^CD8^+^ T cells (Tc1). (**C**) TNF-α expressing CD3^+^CD8^+^ T cells (Tc1). TNF-α expressing CD3^+^CD8^+^ T cells significantly decreased on D21 and D42 compared to D0. (**D**) IL4 expressing CD3^+^CD8^+^ T cells (Tc2). (**E**) IL5 expressing CD3^+^CD8^+^ T cells (Tc2). (**F**) IL13 expressing CD3^+^CD8^+^ T cells (Tc2). (**G**) IL17 expressing CD3^+^CD8^+^ T cells (Tc17). (**H**) IL21 expressing CD3^+^CD8^+^ T cells (Tc17). IL21 expressing CD3^+^CD8^+^ T cells significantly decreased on D21 and D42 compared to D0. (**I**) Comparison of US and STI results. There were no significant differences between the results of CD3^+^CD8^+^ T cells in US and STI groups. However, (**J**) TNF-α expressing CD3^+^CD8^+^ T cells were significantly higher in the STI group. (**K**) Viability increased significantly during storage. Each dot represents each donor and the line over the dots represents the median of the data. The light blue dots indicate CD8 subpopulations (**A**–**H**). The white dots indicate US results, while the dark blue dots indicate STI results (**I**,**J**). The purple dots indicate viability (**K**). Dots aligned with the “0” value on the Y-axis are below the assay’s detection limit. D0: Day 0; D21: Day 21; D42: Day 42; STI: Stimulated; US: Unstimulated; Tc: CD3^+^CD8^+^ cytotoxic T cells. * *p* < 0.05, ** *p* < 0.005, *** *p* < 0.0005.

The frequencies of CD3^+^CD8^+^ T lymphocytes, along with key functional subsets, significantly declined during the 42-day storage of RBC units (*p* < 0.05) (Figure 3A, Table S1). Specifically, the proportions of CD3^+^CD8^+^ T cells producing TNF-α or IL-21 were significantly reduced on D21 and D42 compared to D0 (*p* < 0.05; Figure 3C,H, Table S1).

Other cytokine-producing subsets (IFN-γ^+^, IL-4^+^, IL-5^+^, IL-13^+^, IL-17^+^) were very low on D0 and became undetectable later, precluding a statistical analysis (Figure 3B,D–G, Table S1).

Mitogen stimulation did not alter the overall amount of CD8^+^ T cells (Figure 3J). However, the STI group showed a higher baseline percentage of TNF-α^+^ cells compared to the US group (*p* < 0.05; Figure 3K, Table S1).

In contrast to the loss of cells, overall viability increased progressively throughout storage, with significant increments from D0 to D21 and from D21 to D42 (*p* < 0.05; Figure 3L, Table S1).

### 3.2. Transcription Factors

**Figure 4:**
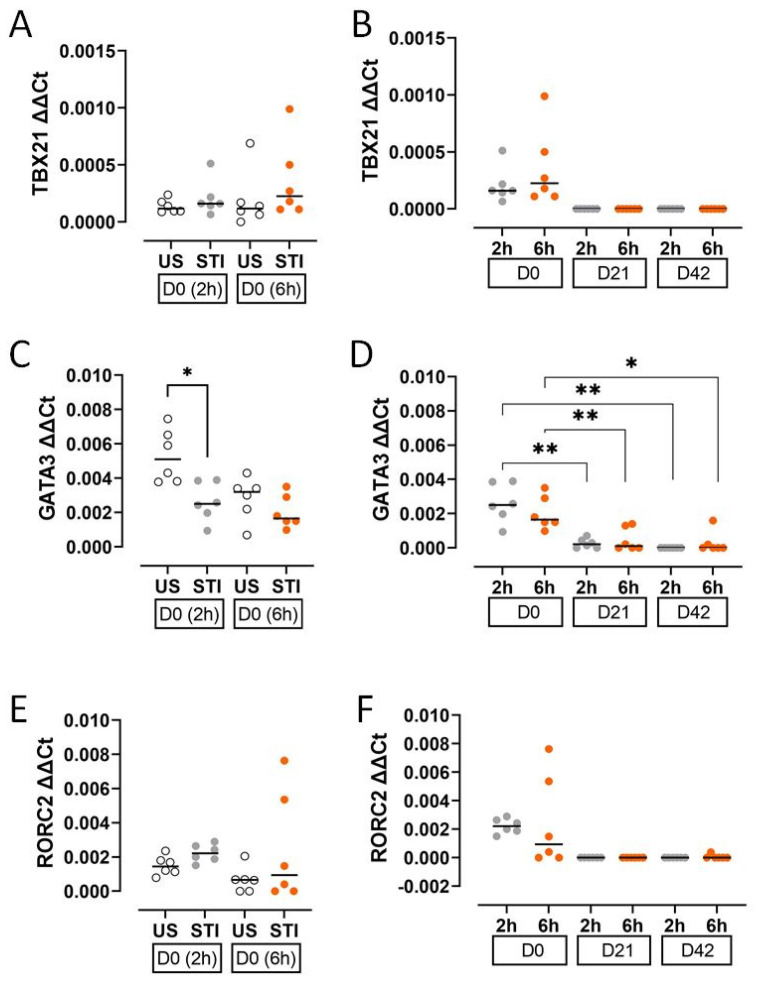
Results and statistical evaluation of transcription factors. (**A**,**B**) TBX21, (**C**,**D**) GATA3, and (**E**,**F**) RORC2. GATA3 was significantly lower in the STI group on D0 (**C**). The levels of transcription factors decreased during storage (**B**,**D**,**F**). The significant decreases in GATA3 levels on D21 and D42 compared to D0 were significant (**D**). Each dot represents each donor and the line over the dots represents the median of the data. The white dots indicate US results, while the grey dots indicate 2 h STI results, and the orange dots indicate 6 h STI results. Dots aligned with the “0” value on the Y-axis are below the assay’s detection limit. D0: Day 0; D21: Day 21; D42: Day 42. * *p* < 0.05, ** *p* < 0.005.

The expression of T cell lineage-defining transcription factors decreased during storage. GATA3 expression was significantly lower in the STI group at an early timepoint (2 h) on D0 compared to the US group (*p* < 0.05; Figure 4C, Table S2).

During storage, levels of TBX21, GATA3, and RORC2 declined markedly at both the 2 h and 6 h timepoints. The decrease in GATA3 was statistically significant (*p* < 0.05; Figure 4D, Table S2), while TBX21 and RORC2 became undetectable on D21 and D42, preventing formal analysis (Figure 4B,F).

### 3.3. Cytokines

Cytokine secretion profiles, reflecting cellular findings, indicated an initial response to the mitogen that was abolished by storage.

**Figure 5:**
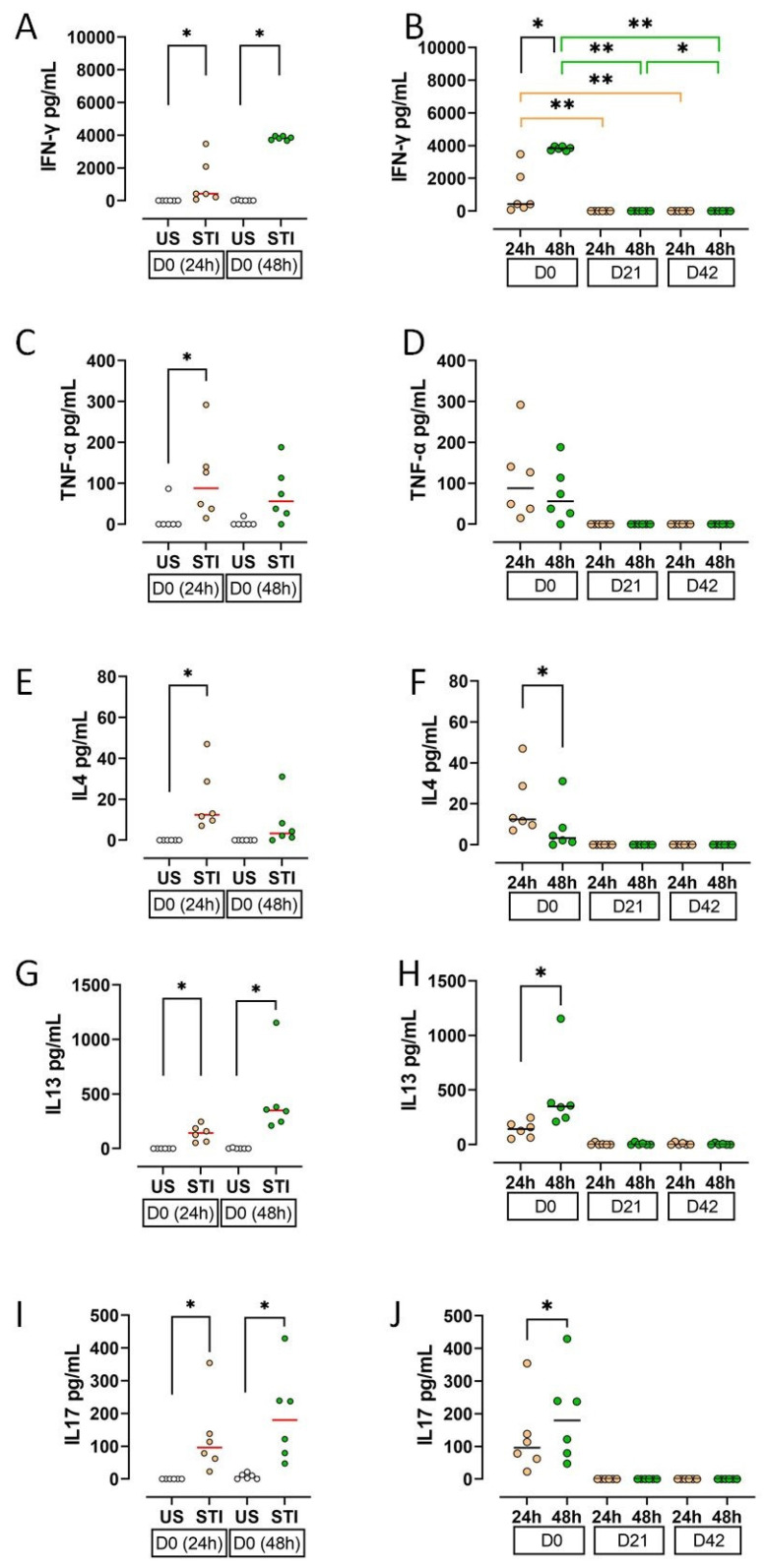
Results and statistical evaluation of cytokines. (**A**,**B**) IFN-γ, (**C**,**D**) TNF-α, (**E**,**F**) IL4, (**G**,**H**) IL13, (**I**,**J**) IL17. IFN-γ, IL13, and IL17 levels were higher in both 24th and 48th hour groups, whereas TNF-α and IL4 were higher only in the 24th hour group (**A**,**C**,**E**,**G**,**I**). All cytokines decreased during storage. The decrease in IFN-γ levels on D21 and D42 compared to D0 was significant (**B**). In addition, IFN-γ, IL13, and IL17 increased significantly at 48 h compared to 24 h (**B**,**H**,**J**), while IL-4 decreased (**F**). Each dot represents each donor and the line over the dots represents the median of the data. The white dots indicate US results, while the light orange dots indicate 24 h STI results, and the green dots indicate 48 h STI results. Dots aligned with the “0” value on the Y-axis are below the assay’s detection limit. D0: Day 0; D21: Day 21; D42: Day 42. * *p* < 0.05, ** *p* < 0.005.

On D0, supernatant levels of IFN-γ, TNF-α, IL-4, IL-13, and IL-17 were significantly higher in STI groups compared to US groups (*p* < 0.05; Figure 5A,C,E,G,I; Table S3). This effect was lost during storage.

All measured cytokines showed a substantial decline on D21 and D42 (Figure 5B,D,F,H,J). The decrease in IFN-γ was statistically significant (*p* < 0.05).

A time-course analysis on D0 revealed that the secretion of IFN-γ, IL-13, and IL-17 increased from the 24th hours to 48th hours, while IL-4 decreased (*p* < 0.05; Figure 5B,F,H,J; Table S3).

## 4. Discussion

Allogeneic MNCs in the blood component are considered as a main inducing factor of TRIM [2]. Previous studies had focused on T lymphocytes in RBC bags. In these studies, CD3^+^CD4^+^ and CD3^+^CD8^+^ T cell counts, viability, and proliferative capacities were found to decrease during the storage time [15,16,17]. Similarly, CD8^+^ T lymphocytes and their subgroups decreased on D21 and D42 in the current study. Most CD3^+^CD8^+^ cells were CD3^+^CD8^+^TNF-α^+^ and CD3^+^CD8^+^IL-21^+^ cells at D0 and decreased during storage. CD3^+^CD8^+^ cells secreting IFN-γ, IL-4, IL-5, IL-13, and IL-17 were very low on D0, D21, and D42. Unlike the previous study, viability increased during storage time in the current study [16]. However, it was consistent with another study indicating that PHA stimulation increases viability [18].

It was also found that CD8^+^ T lymphocytes did not respond to mitogen stimulation (PHA) in the blood bag during storage. CD8^+^ T cell subgroups were not affected by stimulation except CD3^+^CD8^+^TNF-α^+^ on D0. This early activation of CD3^+^CD8^+^TNF-α^+^ cells was abolished during the storage time. This inability to respond may be due to proliferation and viability issues. However, viability increased during storage time. Thus, the decreased proliferative capacity may play an essential role in this outcome. Our previous study showing that CD8^+^ cells lose their proliferative ability during storage supports this idea [16]. Different variables associated with storage conditions and time might have the potential to suppress T cell proliferation. For example, some studies have shown that 4-day storage at 4 °C [19], and prolonged storage of erythrocytes [20,21] can suppress T cell proliferation, including autologous cells. However, this was not observed in fresh red blood cells [20,21]. Furthermore, while the existence of some erythrocyte membrane molecules can provoke the proliferation of CD8^+^ T cells, their absence can lead to suppression [22]. These studies indicate that stored erythrocytes can acquire a suppressive effect on CD8^+^ T cells. Our findings are consistent with the literature suggesting that stored erythrocytes can acquire immunomodulatory properties. Our unresponsive CD8^+^ T cells may have been affected by this kind of erythrocytes, which were affected by the storage condition of the blood bags. More importantly, the T lymphocyte unresponsiveness becoming stronger during storage time was not recovered in ex vivo cell culture. This result also suggests that storage conditions may lead to metabolic inactivity or anergy rather than cell death. Live CD8^+^ T cells may have become functionally impaired, differentiated into cells that cannot proliferate or produce cytokines. This discrepancy between viability and functionality could explain why live lymphocytes in the blood bag did not function effectively in the transfusion recipient. As a result, storage time and conditions appear to play an important role in the suppression and unresponsiveness of CD8^+^ T cells.

The results of the CD8^+^ T cell subgroup were found to be consistent with their specific transcription factors and cytokines. Cytokines and transcription factors decreased during storage time similarly to CD8^+^ T cells. This can be another sign for the importance of the storage time. D0 cytokines may be produced by CD8^+^ T cells that were not affected by storage conditions yet. However, on D0, there were differences between the supernatant (extracellular) and the intracellular cytokine levels. IL-4, IL-13, IL-17, and IFN-γ levels in the supernatant were clearly higher than intracellular levels. This discrepancy may be due to the time of our analysis. It is possible that the intracellular staining was performed after CD8^+^ T cells secreted their cytokines into the supernatant. Such a contradiction was not found between TNF-α expressing cells and TNF-α levels in the supernatant. This may be due to the mitogen stimulation. CD8^+^ T cells expressing TNF-α were the only cells which responded to the stimulation and produced TNF-α until the 48th hour.

TRIM-related effects such as increased renal allograft survival, cancer recurrence, and viral infection relapse seem to be related to the depression of CD8^+^ T cells in transfusion recipients. In contrast, some studies showed that the CD8^+^ counts increase in recipients after ABT. However, the fact that a similar result was obtained after autologous transfusion suggests that the increase is related to the underlying disease [5,23]. Our study has indicated that CD8^+^ T cells that are numerically and functionally diminished by storage enter a state of unresponsiveness which is not reversed by short-term culture. Also, CD8^+^ T cells have lost their viability and proliferative capacity during storage time [15,16,17]. Results of our study support our hypothesis that the factors that trigger TRIM in ABT recipients may also have a similar effect on the cells in blood components. However, it is important to note that some studies have shown no correlation between storage conditions and TRIM [24].

Our ex vivo findings have important implications for TRIM. If transfused, CD8^+^ T cells are hypo-functional and unable to respond to stimulation; their direct contribution to pro-inflammatory or alloimmune reactions in the recipient seems unlikely. The identified loss of CD8^+^ T cell functions shows a consequence of the storage lesion rather than a cause of TRIM. This interpretation shifts the focus of TRIM aetiology to other mechanisms, such as soluble mediators released from stored blood components, storage time, and conditions. Moreover, variables related to blood donors may also have an impact.

Limitations of our study: This study was conducted in an ex vivo model that cannot fully replicate the complex immune environment of the transfusion recipient. Furthermore, the use of a potent mitogen (PHA), which is maximally stimulatory, may differ from responses to physiologically more relevant antigens. Additionally, it was not tested whether the same effects can be observed in the leukoreduced RBCs, because there would not be enough cells remaining in the blood bag after leukoreduction for the experiment. Moreover, the fact that all of our donors were males may also influence our results.

## 5. Conclusions

This study demonstrates that long-term storage of red blood cells leads to a significant numerical and functional decrease in CD8^+^ T lymphocytes. These cells lose their characteristic cytokine profiles and transcription factor expression, becoming insensitive to mitogenic stimulation, which could not be reversed by short-term culturing. The concurrent increase in viability highlights an issue between cellular integrity and immunological function. Our ex vivo findings suggest that transfused CD8^+^ T cells are unlikely to be primary mediators of TRIM. Their functional impairment appears to be part of “storage damage”. Future research should focus on identifying the precise factors inducing this suppressive phenotype in stored blood and elucidating their role in clinical TRIM outcomes.

## Figures and Tables

**Table 1:** CD8^+^ T cell-related cytokines and transcription factors.

Tc Cells	Transcription Factors	Effector Cytokines
Tc1	TBX21	IFN-γ, TNF-α
Tc2	GATA3	IL-4, IL-5, IL-13
Tc17	RORC2	IL-17, IL-21

Tc: CD3^+^CD8^+^ cytotoxic T cells.

## Data Availability

The data presented in this study are available on request from the corresponding author.

## Supplementary Materials

The following supporting information can be downloaded at https://www.mdpi.com/article/10.3390/jcm15031178/s1, Table S1: The levels of CD8^+^ T lymphocytes, their subgroups, and the viabilities. Table S2: The levels of specific transcription factors of CD8^+^ T lymphocytes. Table S3: The levels of effector cytokines of CD8^+^ T lymphocytes.

## Abbreviations

The following abbreviations are used in this manuscript:

ABT	Allogeneic blood transfusion
cDNA	Complementary DNA
D0	Day 0
D21	Day 21
D42	Day 42
ELISA	Enzyme-linked immunosorbent assay
FC	Flow-cytometer
FSC	Forward scatter
mAb	Fluorescent-labelled monoclonal antibody
MNC	Mononuclear cell
PHA	Phytohemagglutinin
RBC	Red blood cell concentrate
RT-PCR	Real-time polymerase chain reaction
SSC	Side scatter
STI	Stimulated
Tc	CD3^+^CD8^+^ cytotoxic T cell
TRIM	Transfusion-related immunomodulation
US	Unstimulated
