# Supplementary material for: Ex Vivo Evaluation of CD3+CD8+ T Cell Subpopulations in Red Blood Cell Concentrates: Does Storage Time Play an Important Role?
Source: J Clin Med. 2026 Feb 3;15(3):1178. doi: 10.3390/jcm15031178 (PMC12897617; doi:10.3390/jcm15031178)
Supplement: Supplementary file 1 [file jcm-15-01178-s001.zip › jcm-4082388-supplementary.pdf]

Supplementary Materials:

Table S1. The levels of $CD8^+$ T lymphocytes, their subgroups, and the viabilities.

		Day 0 (US)	Day 0 (STI)	Day 21 (US)	Day 21 (STI)	Day 42 (US)	Day 42 (STI)
$CD3^+CD8^+$	Med.	29,3%	26,6%	4,7%	5,1%	3,1%	4,2%
	Min.	14,8%	16,8%	3,1%	2,3%	1,6%	1,2%
	Max.	42,9%	42,4%	17,2%	17,9%	7,2%	8,4%
$CD3^+CD8^+TNF^+$	Med.	41,0%	53,5%	1,5%	2,8%	2,0%	0,4%
	Min.	23,6%	43,7%	0,0%	0,0%	0,0%	0,0%
	Max.	52,2%	90,1%	5,1%	11,5%	4,8%	10,9%
$CD3^+CD8^+IFN\text{-}\gamma^+$	Med.	0,1%	1,7%	0,0%	0,0%	0,0%	0,0%
	Min.	0,0%	0,1%	0,0%	0,0%	0,0%	0,0%
	Max.	3,6%	9,6%	0,2%	0,3%	0,0%	0,0%
$CD3^+CD8^+IL\text{-}4^+$	Med.	0,6%	0,4%	2,1%	1,5%	0,7%	0,6%
	Min.	0,4%	0,1%	0,2%	0,7%	0,0%	0,0%
	Max.	1,3%	2,3%	7,2%	7,7%	3,3%	1,8%
$CD3^+CD8^+IL\text{-}5^+$	Med.	6,2%	3,3%	0,8%	1,2%	0,9%	0,5%
	Min.	0,7%	0,1%	0,0%	0,0%	0,0%	0,0%
	Max.	45,5%	36,0%	14,2%	27,6%	5,0%	4,9%
$CD3^+CD8^+IL\text{-}13^+$	Med.	5,8%	1,6%	0,7%	1,2%	0,4%	0,0%
	Min.	0,7%	0,5%	0,0%	0,5%	0,0%	0,0%
	Max.	25,2%	31,0%	9,8%	3,8%	4,3%	5,4%
$CD3^+CD8^+IL\text{-}17^+$	Med.	1,0%	1,9%	1,6%	2,0%	0,6%	0,7%
	Min.	0,4%	1,5%	0,3%	0,0%	0,0%	0,3%
	Max.	1,3%	2,9%	4,3%	8,2%	2,0%	7,8%
$CD3^+CD8^+IL\text{-}21^+$	Med.	32,7%	34,4%	2,5%	3,9%	0,8%	6,9%
	Min.	8,5%	13,9%	0,7%	0,3%	0,0%	0,0%
	Max.	43,5%	82,8%	5,0%	5,8%	3,1%	18,1%
Total Viability	Med.	67,8%	48,1%	81,3%	81,3%	95,1%	96,2%
	Min.	61,0%	31,4%	29,9%	23,8%	68,0%	66,6%
	Max.	71,9%	71,5%	96,4%	95,8%	99,6%	99,7%

US: unstimulated, STI: stimulated

Table S2. The levels of specific transcription factors of $CD8^+$ T lymphocytes.

			Day 0 (US)	Day 0 (STI)	Day 21 (US)	Day 21 (STI)	Day 42 (US)	Day 42 (STI)
TBX21 (ΔΔCt)	2 hour	Med.	1,17E-04	1,61E-04	0,00E+00	0,00E+00	0,00E+00	0,00E+00
		Min.	8,77E-05	6,60E-05	0,00E+00	0,00E+00	0,00E+00	0,00E+00
		Max.	2,38E-04	5,12E-04	0,00E+00	0,00E+00	0,00E+00	0,00E+00
	6 hour	Med.	1,15E-04	2,28E-04	0,00E+00	0,00E+00	0,00E+00	0,00E+00
		Min.	0,00E+00	1,13E-04	0,00E+00	0,00E+00	0,00E+00	0,00E+00
		Max.	6,91E-04	9,94E-04	0,00E+00	0,00E+00	0,00E+00	0,00E+00
GATA3 (ΔΔCt)	2 hour	Med.	5,11E-03	2,51E-03	5,62E-04	2,20E-04	0,00E+00	0,00E+00
		Min.	3,78E-03	9,39E-04	0,00E+00	0,00E+00	0,00E+00	0,00E+00
		Max.	7,46E-03	3,89E-03	1,11E-02	7,09E-04	1,16E-04	0,00E+00
	6 hour	Med.	3,19E-03	1,67E-03	3,18E-04	1,12E-04	0,00E+00	0,00E+00
		Min.	6,91E-04	9,94E-04	0,00E+00	0,00E+00	0,00E+00	0,00E+00
		Max.	4,26E-03	3,45E-03	2,65E-03	1,38E-03	0,00E+00	1,57E-03
RORC2 (ΔΔCt)	2 hour	Med.	1,45E-03	2,22E-03	0,00E+00	0,00E+00	0,00E+00	0,00E+00
		Min.	7,91E-04	1,51E-03	0,00E+00	0,00E+00	0,00E+00	0,00E+00
		Max.	2,36E-03	2,89E-03	0,00E+00	0,00E+00	0,00E+00	0,00E+00
	6 hour	Med.	6,64E-04	9,43E-04	0,00E+00	0,00E+00	0,00E+00	0,00E+00
		Min.	0,00E+00	0,00E+00	0,00E+00	0,00E+00	0,00E+00	0,00E+00
		Max.	2,05E-03	7,63E-03	3,89E-04	0,00E+00	0,00E+00	3,79E-04

US: unstimulated, STI: stimulated

Table S3. The levels of effector cytokines of CD8+ T lymphocytes.

			Day 0 (US)	Day 0 (STI)	Day 21 (US)	Day 21 (STI)	Day 42 (US)	Day 42 (STI)
**IL-4 (pg/mL)**	24 hour	Med.	0,0	12,4	0,0	0,0	0,0	0,0
		Min.	0,0	7,1	0,0	0,0	0,0	0,0
		Max.	0,0	47,0	0,0	0,0	0,0	0,0
	48 hour	Med.	0,0	3,3	0,0	0,0	0,0	0,0
		Min.	0,0	0,0	0,0	0,0	0,0	0,0
		Max.	0,0	31,1	0,0	0,0	0,0	0,0
**IL-13 (pg/mL)**	24 hour	Med.	0,0	142,5	0,0	0,3	0,0	1,1
		Min.	0,0	54,1	0,0	0,0	0,0	0,0
		Max.	0,0	246,2	5,6	26,0	13,3	26,9
	48 hour	Med.	0,0	349,8	0,0	0,0	0,0	0,0
		Min.	0,0	210,5	0,0	0,0	0,0	0,0
		Max.	9,9	1153,8	6,5	27,7	19,2	18,4
**IL-17 (pg/mL)**	24 hour	Med.	0,0	96,2	0,0	0,0	0,0	0,0
		Min.	0,0	23,0	0,0	0,0	0,0	0,0
		Max.	0,0	354,3	0,0	0,0	0,0	0,0
	48 hour	Med.	6,2	179,8	0,0	0,0	0,0	0,0
		Min.	0,0	47,4	0,0	0,0	0,0	0,0
		Max.	22,1	429,3	0,0	0,0	0,0	0,0
**TNF-α (pg/mL)**	24 hour	Med.	0,0	88,0	0,0	0,0	0,0	0,0
		Min.	0,0	15,1	0,0	0,0	0,0	0,0
		Max.	86,8	292,0	0,0	0,0	0,0	0,0
	48 hour	Med.	0,0	56,0	0,0	0,0	0,0	0,0
		Min.	0,0	0,0	0,0	0,0	0,0	0,0
		Max.	20,1	188,3	0,0	0,0	0,0	0,0
**IFN-γ (pg/mL)**	24 hour	Med.	11,2	427,1	6,5	4,4	1,8	2,3
		Min.	7,6	74,9	2,3	2,3	0,0	0,0
		Max.	14,9	3481,4	9,7	5,5	6,5	5,5
	48 hour	Med.	14,4	3833,6	4,4	5,5	4,4	2,3
		Min.	10,7	3675,9	2,3	0,2	0,0	0,0
		Max.	70,6	3962,9	6,5	7,6	7,6	3,4

US: unstimulated, STI: stimulated
